# Authenticity, acceptability, and feasibility of a hybrid gynecology station for the Papanicolaou test as part of a clinical skills examination in Korea

**DOI:** 10.3352/jeehp.2018.15.4

**Published:** 2018-02-13

**Authors:** Ji-Hyun Seo, Younglim Oh, Sunju Im, Do-Kyong Kim, Hyun-Hee Kong, HyeRin Roh

**Affiliations:** 1 Department of Pediatrics and Medical Education, Gyeongsang National Institute of Health Sciences, Gyeongsang National University School of Medicine, Jinju, Korea; 2 Department of Obstetrics and Gynecology, Kosin University College of Medicine, Busan, Korea; 3 Department of Medical Education, Pusan National University School of Medicine, Busan, Korea; 4 Department of Medical Humanities, Dong-A University College of Medicine, Busan, Korea; 5 Department of Parasitology, Dong-A University College of Medicine, Busan, Korea; 6 Department of Medical Education and the Institute for Medical Humanities, Inje University College of Medicine, Busan, Korea; Hallym University, Korea

**Keywords:** Clinical competence, Papanicolaou test, Medical students, Patient simulation, Korea

## Abstract

**Purpose:**

The objective of this study was to evaluate the authenticity, acceptability, and feasibility of a hybrid station that combined a standardized patient encounter and a simulated Papanicolaou test.

**Methods:**

We introduced a hybrid station in the routine clinical skills examination (CSE) for 335 third-year medical students at 4 universities in Korea from December 1 to December 3, 2014. After the tests, we conducted an anonymous survey on the authenticity, acceptability, and feasibility of the hybrid station.

**Results:**

A total of 334 medical students and 17 professors completed the survey. A majority of the students (71.6%) and professors (82.4%) agreed that the hybrid station was more authentic than the standard CSE. Over 60 percent of the students and professors responded that the station was acceptable for assessing the students’ competence. Most of the students (75.2%) and professors (82.4%) assessed the required tasks as being feasible after reading the instructions.

**Conclusion:**

Our results showed that the hybrid CSE station was a highly authentic, acceptable, and feasible way to assess medical students’ performance.

## Introduction

Standardized patient (SP)-based clinical skills examinations (CSEs) have been widely used to assess the clinical performance of medical students or residents in the context of clinical education. Although SPs can skillfully simulate patients’ symptoms and signs in training sessions for intimate examination skills [1], we cannot allow examinees to perform intimate examinations as part of CSEs due to difficulties such as the presence of too many examinees in Korea [2], potential unsafety for the SPs, and inability of the SPs to simulate real pathologic signs in the pelvis. Thus, we have usually given written descriptions of intimate examination findings to examinees in the context of an SP encounter. This does not reflect the reality of clinical encounters (i.e., this method lacks authenticity), and it fails to assess students’ true competency in performing relaxed and patient-centered intimate examinations as part of patient encounters.

Intimate examinations, such as pelvic exams, should be assessed in an authentic way because that is the only way to assess students’ real ability to deal with gynecologic patients. According to the relevant literature, a few hybrid stations have been developed for technical skills assessment [3], but no study has been found on a hybrid CSE station simultaneously assessing history-taking, a physical examination including an intimate examination, and patient-physician interactions. In this context, we developed a hybrid SP station using SPs and gynecologic models of a Papanicolaou (Pap) test. We present our trial of hybrid CSE station implementation and an evaluation of the authenticity, acceptability, and feasibility of the hybrid SP-based gynecology CSE station. We hypothesized that the medical students would evaluate the hybrid station as being more authentic, acceptable, and feasible than the other SP encounter stations.

## Case presentation

### Ethical statement

All data on the scores and results of the survey were provided by the Busan-Gyeongnam Clinical Skills Examination Consortium (BGCSEC) after approval by the institutional review board of Gyeongsang National University (IRB approval no., GIRB-G15-X-0056). The board exempted the study from the requirement for informed consent because of the retrospective nature of the research.

### Case

The study participants were 335 third-year students, comprising examinees of 4 universities, and 22 professors who served as raters and were recruited from 5 universities in the BGCSEC. The joint CSE was composed of 6 technical skills and 6 SP-based stations.

We developed a hybrid case using a Pap test with a scenario and an evaluation form after 6 sessions of 3-hour workshops during 3 months from June 1 to August 31, 2014. The patient in the case was a 45-year-old woman with vaginal bleeding. The medical students’ task at this station was to interview the SP, to perform a focused physical examination, and to write an appropriate entry in the patient’s medical record (Appendix 1). We instructed students to perform an intimate examination on a pelvic model that was provided. The hybrid station took 15 minutes, 5 minutes longer than other SP stations.

**Fig. 1.**
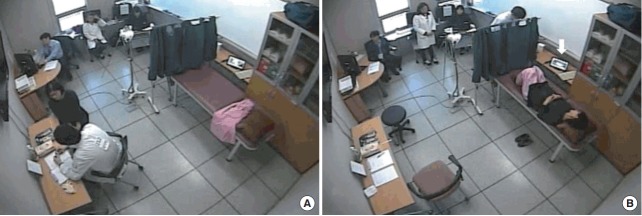
(A) A student interviewing the SP while performing the hybrid clinical skills examination. (B) The SP lying on the bed for a pelvic examination. A computer monitor on the next table (white arrow) is recording the student’s performance, and the SP is observing the student’s performance via a computer monitor behind the curtain. SP, standardized patient.

**Fig. 2.**
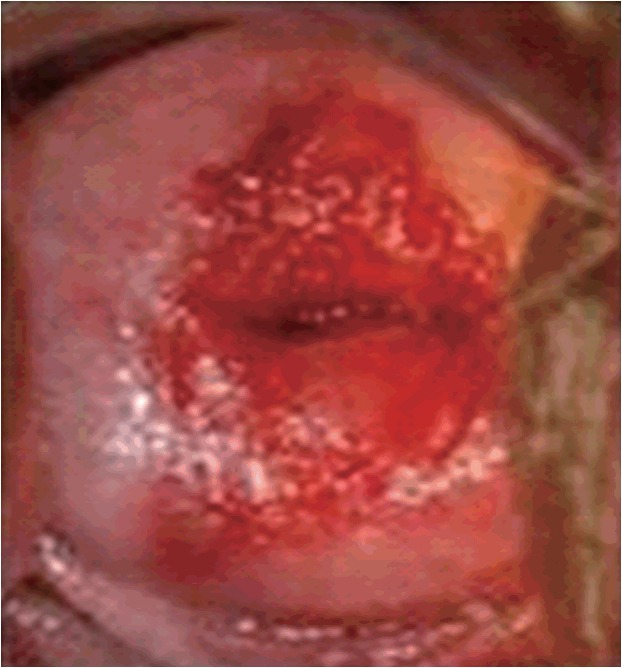
This picture of cervicitis was pasted on the pelvic model to simulate a lesion on the cervix.

We created a station room that resembled a real clinical setting. The SP was first interviewed by the student, and then lay on the bed for a physical examination (Fig. 1A). A nurse curtained off the table to separate the pelvic model from the SP. The simulated pelvic model had a specially designed lesion on the cervix, which was intended to be the primary examination finding for the real patient (Fig. 2). A small camera connected to a computer was set up to capture the performance of the student. While a student explained the pelvic examination, the SP pretended to change clothes and the nurse placed curtains in front of the pelvic model. The SP observed the student’s performance via a computer behind the curtain (Fig. 1B) and responded in a realistic fashion if the student performed uncomfortable procedures, such as abrupt insertion and widening of a speculum.

The items used to assess students’ competency consisted of 5 categories: history-taking, physical examination, clinical reasoning, patient education, and communication skills (Appendix 2). The scores were allocated as 30 points for history-taking, 30 points for the physical examination, 10 points for clinical reasoning, 10 points for patient education, and 20 points for communication skills, resulting in a total of 100 points. Professors rated all the items, except communication skills, which were rated by the SP.

We conducted a pilot study with 20 fourth-year medical students at 1 university, based on which we finalized the scenario and assessment items with several revisions.

We developed a survey questionnaire to evaluate the authenticity, acceptability, and feasibility of the hybrid station, with a rubric according to which each item was scored on a 5-point Likert scale. We administered the questionnaire in written form immediately after the exams were finished. We conducted a descriptive statistical analysis, including the frequency and percentage of answers, using IBM SPSS ver. 21.0 for Windows (IBM Corp., Armonk, NY, USA).

The average total CSE score for the other 5 stations was 58.0± 7.7 points (maximum score, 65.3 points), whereas that of the hybrid station was 60.32± 10.8 points, showing no statistically significant difference. The internal consistency for the 18 items in the hybrid station was 0.779 (Cronbach alpha), and the other 5 SP stations had similar internal consistency results (range, 0.690–0.878). A total of 334 medical students (99.7%) and 17 professors (77.3%) completed the survey. A majority of the medical students (71.6%) and professors (82.4%) agreed that the hybrid station was more authentic than the standard CSE (Tables 1, 2). More students (64.7%) than professors (41.2%) found it difficult to carry out the required tasks at the hybrid station. A majority of medical students (63.9%) and most professors (94.1%) responded that the task was acceptable for assessing the students’ competence.

A majority of the medical students (75.2%) and professors (82.4%) considered the tasks to be feasible after reading the instructions for the hybrid station. More than half of the students (55.2%) agreed that an appropriate amount of time was allotted for the hybrid station, as did 76.5% of the professors. The raw data are available in Supplement 1.

## Discussion

This is the first trial to be reported in the literature of a hybrid CSE combining an SP clinical encounter and a technical skills station. In standard gynecologic SP stations, students have to stop the interaction with the SP while reading a written description of the pelvic examination. The general technical skills assessment only assesses whether students perform the procedures of the Pap test accurately. However, in this study, we sought to assess students’ ability to perform the entire process of a gynecologic patient encounter, including an intimate examination without a written description.

Forsaking the standard gynecologic stations, where the SP gives a written description of the examination findings to students, we assessed students in a realistic clinical setting in which they performed a Pap test on a pelvic model. Most of the students and professors agreed that the hybrid station was more realistic and practical than the standard CSE, proving the authenticity of the hybrid station. Both real and simulated patient involvement have been found to show positive impacts on the assessment of intimate examination skills [1]. Therefore, we suggest that this hybrid station is a more useful and safer way to assess students’ pelvic examination skills.

Most of the medical students (63.9%) and professors (94.1%) agreed that the task was acceptable as a way to assess the students’ competence. The average score of the hybrid station was similar to those of the other 5 SP stations, further suggesting that this hybrid station was acceptable for assessing the students’ clinical reasoning and communication skills at the same time.

Three-fourths of the medical students and most of the professors agreed that the tasks were feasible after reading the instructions for the hybrid station, and more than half of the students and three-fourths of the professors agreed that a proper amount of time was allotted for the hybrid station. The fact that the average score of the hybrid station was similar to those of other stations also supports the conclusion that this 15-minute hybrid station was an acceptable tool for assessing students’ performance.

We set up this station to realistically simulate outpatient gynecology clinics, and students could see the pelvic model when they entered the room. Therefore, all students tried to perform the Pap test, which may have tended to increase their total scores at the hybrid station, although 64.7% of the students felt that the hybrid station was more difficult than the other SP encounter stations. Students have been found to be nervous in the presence of SPs, leading to lower scores on pelvic exams [4]. A more detailed and well-articulated scenario, as well as variations in the station setup, space, and checklist, should be developed to assess whether students make different diagnoses and perform different tests for different patients when similar test items are present in the exams. Various hybrid stations should be developed that will be helpful for assessing medical students’ communication skills, ethics, and technical skills. Further investigations should focus on the impact of hybrid stations on students’ attitudes towards various responses of the SPs.

The limitations of the present study are as follows: the retrospective cross-sectional observational design, and the use of only 1 hybrid station as the comparison group. Nevertheless, we found that using an SP combined with a simple skills test was an authentic, acceptable, and feasible way of assessing students’ performance of both technical skills and clinical reasoning skills.

In conclusion, our results satisfied the hypothesis that medical students would evaluate the hybrid station as being more authentic, acceptable, and feasible than the other SP encounter stations. Therefore, we suggest that hybrid exams are a useful tool for assessing students’ performance, including clinical reasoning ability.

## Figures and Tables

**Table 1.** Survey of students regarding the authenticity, acceptability, and feasibility of the hybrid station

Variable	Excellent (%)	Good (%)	Average (%)	Mediocre (%)	Poor (%)
Authenticity					
Realistic compared to classical gynecologic SP-based tests	18.3	53.3	25.4	2.1	0.9
Acceptability					
Difficult compared to classical SP-based tests	18.6	46.1	32	3.3	0
Appropriate for assessing a student’s competence	11.6	52.3	31.3	1.8	0.7
Feasibility					
Identifying the task after reading the instructions	13.8	61.4	22.8	1.8	0.3
The duration of the test was appropriate	11.1	44.1	30.6	13.2	1.0

SP, standardized patient.

**Table 2.** Survey of professors on the authenticity, acceptability, and feasibility of the hybrid station

Variable	Excellent (%)	Good (%)	Average (%)	Mediocre (%)	Poor (%)
Authenticity					
Realistic compared to classical gynecologic SP-based tests	5.9	76.5	17.6	0	0
Acceptability					
Difficult compared to classical SP-based tests	5.9	35.3	47.1	11.8	0
Appropriate for assessing a student’s competence	5.9	88.2	5.9	0	0
Feasibility					
Identifying the task after reading the instructions	11.8	70.6	11.8	5.9	0
The duration of the test was appropriate	11.8	64.7	5.9	11.8	5.9

SP, standardized patient.

## Supplementary materials

Supplement 1. Data files are available from https://doi.org/10.7910/DVN/FECNDV

Supplement 2. Audio recording of the abstract
