# Supplementary material for: Authenticity, acceptability, and feasibility of a hybrid gynecology station for the Papanicolaou test as part of a clinical skills examination in Korea
Source: J Educ Eval Health Prof. 2018 Feb 13;15:4. doi: 10.3352/jeehp.2018.15.4 (PMC5900362; doi:10.3352/jeehp.2018.15.4)
Supplement: Supplementary file 2 [file jeehp-15-04-app1.pdf]

Appendix 1. Situation guidelines

A 45-year-old woman (Kim OO, hospital number 14345) visited the hospital after having found blood on her underwear.

< Vital signs >
Blood pressure: 120/80 mm Hg
Pulse rate: 70/min
Respiration rate: 18/min
Body temperature: 36.7℃

The examinee will be given 15 minutes to:

- Take the patient's history of symptoms.
- Perform an appropriate physical examination in relation to the symptoms.
- Perform the necessary tests using the model.
- Discuss the diagnosis and treatment plan with the patient.

Appendix 2. Rubric for assessing a student's performance

Title: vaginal bleeding					
1. History-taking (30%)	Yes		No		
Main symptoms					
Abnormal hormonal or genitourinary condition					
Infection versus tumor					
2. Physical examination (30%)	Properly performed		Partly performed		Poorly performed
Inspection of conjunctivae (degree of bleeding)					
Inspection of cervix and vagina					
Abnormal hormonal or genitourinary condition					
- Pap test					
- Manual pelvic exam					
Proficiency of physical examination	Excellent		Average		Poor
Attitude during the pelvic examination					
3. Clinical reasoning (10%)					
4. Patient education (10%)					
5. Communication skills: patient-physician interaction (20%)	Excellent	Good	Average	Less than average	Poor
Total score (100)					
